# Effects of Different Pretreatments on Hot Air Drying Characteristics, Nutrition, and Antioxidant Capacity of Tartary Buckwheat Sprouts

**DOI:** 10.3390/foods13162473

**Published:** 2024-08-06

**Authors:** Xianmeng Xu, Nan Wang, Shunmin Wang, Junzhen Wang, Ningning Wu, Yudie Xu, Min Xu

**Affiliations:** 1 Department of Biological and Food Engineering, Bozhou University, Bozhou 236800, China; 2 College of Biological and Food Engineering, Anhui Polytechnic University, Wuhu 241000, China; w13721290863@163.com (N.W.); seanning914@163.com (N.W.); xu20240417@163.com (Y.X.); 3 Academy of Agricultural Science, Liangshan 615000, China; wangjunzhen108@163.com

**Keywords:** Tartary buckwheat sprouts, hot air drying, β-cyclodextr, active ingredients

## Abstract

In order to enhance the quality of hot air drying for Tartary buckwheat sprouts and minimize the loss of active substances, this research explored the impact of Color Protection(CP), Osmosis(OM), Blanching (BC), β-cyclodextrin (β-CD), and Ultrasound (US) in conjunction with hot air drying on the color, nutritional value, antioxidant properties, and other attributes of Tartary buckwheat sprouts. The findings revealed that as the drying temperature increased from 50 °C to 70 °C, the drying duration for Tartary buckwheat sprouts decreased across all treatment groups, leading to a higher dehydration rate. Treatments involving CP, US, and BC effectively reduced the drying time of Tartary buckwheat sprouts. Sprouts subjected to CP, β-CD, and US treatments exhibited elevated L* values and decreased a* values and displayed a more vibrant green color. When exposed to a hot air setting of 60 °C, the total flavonoid content in the CP, OM, β-CD, and US groups increased by 8.76%, 6.76%, 12.34%, and 4.25%, respectively, compared to the Control Group (CK). The application of the CP, OM, β-CD, and US treatments enhanced the sprouts’ ability to combat ABTS and DPPH free radicals. Notably, under hot air conditions of 60 °C, the β-CD treatment demonstrated the most effective quality preservation during the hot air drying process for sprouts. This study provides valuable insights into the drying behavior of Tartary buckwheat sprouts and offers guidance for optimizing the drying procedures in industrial settings. Tartary buckwheat sprouts contain a variety of polyphenols and have a high water content. The study of changes in active components such as polyphenols and their alteration mechanisms in Tartary buckwheat sprouts under different processing methods is particularly important for the development of sprout processing.

## 1. Introduction

Tartary buckwheat is a unique crop that combines seven essential nutrients, including 18 natural amino acids, peptides, flavonoids, trace elements, plant sterols, carotenoids, and unsaturated fatty acids, whose global production was 11 million 810 thousand metric tons in 2020 [1]. Due to its impressive nutritional profile, it is often referred to as the “king of grain” [2]. Tartary buckwheat has the “three lowering effects” of lowering blood pressure, lowering blood lipids, and lowering blood sugar, as well as antioxidation, antitumor, antiinflammatory properties in addition to other functions [3]. Tartary buckwheat sprouts grown from Tartary buckwheat seeds have a high flavonoid content, such as rutin, quercetin, vitexin, and isoflavone [4].

Fresh sprouts are healthy foods, but their shelf life is short, so they need to be processed to maintain their nutritional value. Drying is the most common form of food preservation, which improves food stability and minimizes physical and chemical changes during storage [5,6]. However, due to the diversity of polyphenols and various degradation enzymes, the stability of Tartary buckwheat sprouts changes during processing [7]. It leads to the degradation of phenolic substances such as rutin and enzymatic browning, resulting in quality loss and nutritional degradation [8].

Pretreatment technology is a common method for improving drying efficiency, food quality, and the value of products [9]. It includes ultrasonic pretreatment [10], infrared pretreatment [11], and alcohol pretreatment [12]. In order to improve the quality of hot air drying, we often pretreat the products by applying physical, chemical, biological, and other technical means before drying. This approach speeds up the drying rate, improves appearance and nutritional quality, extends shelf life, and allows for the addition of functional ingredients [13]. For example, sodium chloride can effectively maintain the color, texture, and flavor of fruits and vegetables, reduce water activity, and extend shelf life [14]. The contents of total flavonoids and polyphenols in dried daylily products can be increased by a 1–2% citric acid treatment, resulting in improved product color [15]. According to Lavelli et al. [16], blanched carrots showed 51%, 76%, and 87% higher levels of alpha-carotene, beta-carotene, and lutein, respectively, compared to unblanched carrots. Furthermore, another study reported that after subjecting pineapple to a 40 kHz ultrasonic treatment, drying times decreased by 55% at 40 °C and 42% at 70 °C [17],. Treatment with 20% sproutdextrin and β-cyclodextrin has been shown to effectively improve the rehydration rate and product yield of freeze-dried soybean sprouts [18].

In this study, buckwheat sprouts were pretreated for drying using CP, OM, BC, β-CD, and US. The effects of different pretreatments on the hot air drying characteristics of buckwheat sprouts, as well as the color, nutritional value, antioxidant properties, and other attributes of Tartary buckwheat sprouts, and other indicators, were summarized. The study also investigated the effect of pretreatment combined with hot air drying on the drying quality of buckwheat sprouts. It was investigated with the expectation of reducing the loss of flavonoids, total phenols, and other bioactive components during the hot air drying of buckwheat sprouts as well as improving the antioxidant capacity. To improve the quality of Tartary buckwheat sprouts during hot air drying and to minimize active substance loss, this research examined drying processes using temperatures of 50 °C, 60 °C, and 70 °C. It utilized color protection, penetration, blanching, β-cyclodextrin, and ultrasonic pretreatment techniques. The study monitored changes in moisture content, color, rehydration, physical properties, nutrient composition, and antioxidant capacity during the drying process, ultimately determining the optimal drying conditions for achieving the highest quality buds. It is particularly important for the development of sprout processing.

## 2. Materials and Methods

### 2.1. Plant Materials and Chemical Reagents

Tartary buckwheat seeds (Chuanqiao No. 1) were obtained from the Xichang Agricultural Science Research Institute (Liangshan, China) and stored at −20 °C before the experiment. All chemicals (such as citric acid, NaCl,β-cyclodextrin,3,5-dinitrosalicylic acid, coomassie brilliant blue, sodium nitrite, aluminum nitrate, folin-phenol and so on) were analytically pure and purchased from Sinopharm Chemical Reagent (Shanghai, China). Except 2,2′-Azinobis-(3-ethylbenzthiazoline-6-sulphonate) (ABTS) and 2,2-Diphenyl-1-picrylhydrazyl (DPPH) were purchased from Sigma-Aldrich (Merck & Co., Inc., St. Louis, MO, USA).

### 2.2. Sprout Sample Preparation

All Tartary buckwheat seeds were screened and cleaned, sterilized by soaking in 1 g/L potassium permanganate solution for 5 min, soaked in distilled water for 12 h, and then spread on the seedling tray. After two days of germination under conditions of 20 °C and 80% humidity, the seeds were exposed to light for 12 h each day and sprayed with water regularly. The sprouts were collected after 10 days. The collected Tartary buckwheat sprouts were cleaned and rinsed in clear water, and we removed surface water by natural air and stored them in the refrigerator at 4 °C for later use.

### 2.3. Sprouts Pretreatment

According to the results of preliminary experiments, the different pretreatment conditions of Tartary buckwheat sprouts were determined as Table 1.

### 2.4. Hot Air Drying

The pretreated Tartary buckwheat sprouts (50 g) were spread on the hot air drying net with a load capacity of 0.12 g/cm^2^ and placed in hot air drying chambers with temperatures set to 50 °C, 60 °C, and 70 °C. Samples were taken out every 30 min for weighing and dried to a constant weight, which was defined as a change in mass of ±0.002 g within 30 min. Each drying procedure was repeated three times. The obtained dry samples were crushed and passed through an 80-mesh sieve, then the sieved powder was ready for use.

### 2.5. Drying Curve

The dry base moisture content during the hot air drying process of Tartary buckwheat sprouts was calculated according to Equation (1) [9]:

(1) $$M_{t}=\left(m_{t}-m_{d}\right)/m_{d}$$

where *M_t_* is the dry base moisture content at time *t*, g/g; *m_t_* is the mass of the sample at time *t*, g; *m_d_* is a constant mass after drying at 105 °C, g.

### 2.6. Rehydration Ratio

An appropriate amount of dehydrated Tartary buckwheat sprouts was weighed in a 500 mL beaker, and a sufficient amount of boiling water was added. The beaker was sealed with plastic and left at room temperature for 30 min. The sprouts were then drained, and the surface water was removed using filter paper before weighing them again [19]. The rehydration ratio was calculated as the ratio of mass before and after rehydration.

(2) $$M_{R}=m_{1}/m_{0}$$

where *M*_1_ is the quality of Tartary buckwheat sprouts after water absorption, g; *m*_0_ is the quality of Tartary buckwheat sprouts before water absorption, g.

### 2.7. Determination of Color Profile

The freeze-dried samples were measured for 4 times using a portable colorimeter (Konica Minolta, Inc., Tokyo, Japan). The L* (lightness: 0-black, 100-white), a* (+a*-redness, −a*-greenness), and b* (+b*-yellowness, −b*-blueness) of the samples were determined according to the CIELAB color system [20].

### 2.8. Physical Characteristics

The resting angle and sliding angle were measured following the methods of Sun et al. [21], with slight modifications. The packing density of Tartary buckwheat sprout powder (×10^3^ kg/m^3^) was based on the method of Knop and Peled [22]. Water retention and oil retention were measured according to the methods of Zhou et al. [23] and Zhao et al. [24], respectively. For solubility, the Alves et al.’s method [25] was followed with slight modifications.

### 2.9. Nutritional Properties

The reducing sugar content was determined using the 3,5-dinitrosalicylic acid method [4]; the soluble protein content was measured using the Coomassie brilliant blue staining method [4]; the total chlorophyll content was determined using UV-visible spectrophotometric method [26]; the starch content was determined using the iodine-starch colorimetric method [27]; the total flavonoid content was determined using the sodium nitrite and aluminum nitrate color developing methods [28]; the total phenolic content was determined using the Folin-phenol colorimetric method [5]; and the cellulose content was determined according to the method of Diamante et al. [29].

### 2.10. DPPH Radical Scavenging Ability

The DPPH free radical scavenging ability was determined using the method described by Ma et al. [30], with some modifications. The DPPH radical ethanol solution (0.1 mmol, 4.5 mL) was mixed with 0.2 mL of ethanol (70%, *v*/*v*) extract from the samples. After incubation in the dark for 6 min at 25 °C, the absorbance was measured at 517 nm. A mixture of 0.2 mL of 70% (*v*/*v*) ethanol and 3 mL of 0.1 mmol DPPH radical ethanol solution was used as the blank control. The DPPH radical scavenging ability (RSA) was calculated using the following equation:

(3) $$DPPH\;radical\;scavenging\;activity=\left(1-{Abs}_{sample}/{Abs}_{control}\right)\times 100$$

### 2.11. ABTS Radical Scavenging Ability

The ABTS assay was performed according to the procedure described by Fong-in et al. [31] with some modifications. Briefly, equal volumes of 7 mmol ABTS stock solution and 2.45 mmol potassium persulfate solutions were mixed and left to react for 12–16 h in darkness at room temperature. Before use, the ABTS stored solution was thinned with methanol to obtain an absorbance of 0.7 ± 0.02 at 734 nm. For the ABTS measurement, 1.9 mL of the diluted ABTS solution was transferred to 0.1 mL of mushroom extract and mixed, and the absorbance was read at 734 nm after incubation at room temperature in the dark for 6 min. A blank control was prepared by mixing 0.1 mL of 70% (*v*/*v*) ethanol with 1.9 mL of the ABTS reaction solution. The ABTS RSA was calculated using the following formula:

(4) $$ABTS\;radical\;scavenging\;activity=\left(1-{Abs}_{sample}/{Abs}_{control}\right)\times 100$$

### 2.12. FRAP Assay

The ferric-reducing antioxidant power total reducing capacity was determined in accordance with the experimental method of Peng et al. [32], with certain modifications. Pipette 0.3 mL of sample diluted extract and 2.7 mL of FRAP reagent, mix well and place in a 37 °C water bath for 40 min. Measure the absorbance at 595 nm, using distilled water as a blank to zero the spectrophotometer.

### 2.13. Data Processing

Statistical analysis was performed using the SPSS package program version 14.0 (SPSS Inc., Chicago, IL, USA). All data were expressed as the mean ± standard error (SE) and analyzed by one-way analysis of variance (ANOVA). Statistical significance was determined at a level of *p* < 0.05.

## 3. Result and Analysis

### 3.1. Effect of Different Pretreatment on Moisture Curve of Tartary Buckwheat Sprouts Dried by Hot Air

**Figure 1:**
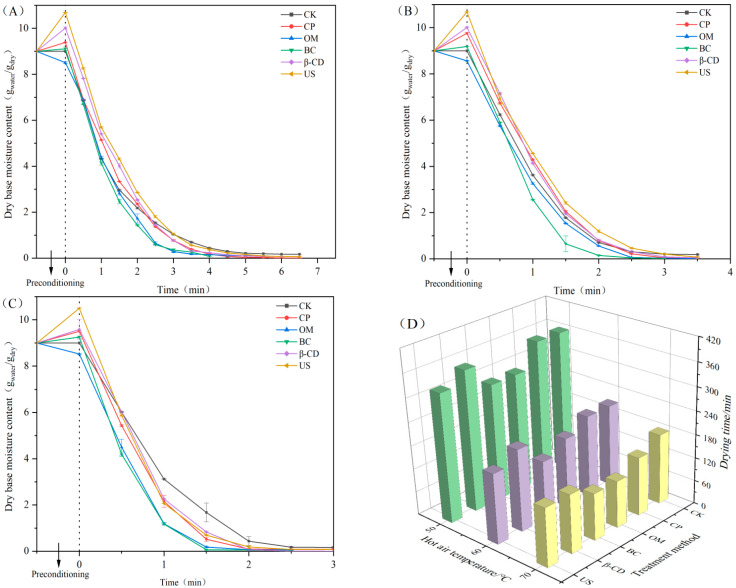
Hot air drying moisture curve and drying time of Tartary buckwheat sprouts under different pretreatment. Note: (**A**) Hot air drying temperature: 50 °C; (**B**) Hot air drying temperature: 60 °C; (**C**) Hot air drying temperature: 70 °C; (**D**) Drying time of the sprouts in each treatment group. Different lowercase letters indicate significant statistical differences (*p* < 0.05).

Figure 1 presents the moisture drying curve of Tartary buckwheat sprouts under different hot air drying temperatures after different pretreatments. At 50 °C, 60 °C, and 70 °C hot air temperatures, the water content of all treatment groups increased during the pretreatment stage, except for the OM group. The sodium chloride infiltration pretreatment created a concentration gradient between the inside and outside of the cell wall, resulting in internal water loss. During the pretreatment stage, the water content of the samples in the US group increased the most due to the destruction of cell tissue in the sprouts and the increase of voids caused by ultrasound, allowing distilled water to easily enter and fill the gaps [33]. The buds of the β-CD group also had higher water content after pretreatment because β-CD itself has higher water retention. It can be concluded that different pretreatments had different effects on Tartary buckwheat sprouts, and their mechanisms were also different. By comparing the changes in water content of Tartary buckwheat sprouts under different hot air temperatures, it can be observed that as the hot air drying temperature increases, the time for the sprouts to reach water balance shortened gradually. High temperatures can accelerate the migration of water inside the sprouts, resulting in faster migration of water molecules from the interior of the sprouts [34].

As shown in Figure 1D, the time for group CK to reach equilibrium moisture content was 360 min at 50 °C, 210 min at 60 °C, and 180 min at 70 °C, respectively. With the increase in hot air temperature, the drying time of group CK gradually shortened. Under different hot air temperature conditions, the OM, BC, and US groups can effectively minimize the drying time, each through different mechanisms. There was no significant difference in drying time between the β-CD and CP groups. Under the same hot air drying temperatures, OM, BC, and US can effectively reduce the drying times of the sprouts, and the drying times at 60 °C and 70 °C were significantly shorter than at 50 °C. The shortest drying time was 120 min for the OM and BC groups under 70 °C hot air, as the tissue structure of the sprouts changed during the pretreatment process, which made the water loss easier and promoted the sprouts to reach the drying balance faster.

### 3.2. Effects of Ultrasound Combined with ZnO NPs Treatment on Colors of Fresh-Cut Lettuce

TAs shown in Table 2, the results showed that at 50 °C, the L* value of Tartary buckwheat sprouts in the CP, β-CD, and US groups increased to 66.05, 66.00, and 65.85, respectively, alleviating the browning of the samples. This increase may be due to beta-cyclodextrin, which can act as a protective agent, spraying on the surface of vegetables to prevent oxidative browning. Citric acid itself acts as an antioxidant, preventing color changes during the sample drying process. Ultrasound speeds up the drying rate, reducing the time of the sample’s exposure to hot air and thereby decreasing the occurrence of non-enzymatic browning [8]. At the same time, the low a* value indicates that the green loss of the samples in the three groups is minimal during the drying process. The a* value is highest in the BC group because the hot process will lead to cell destruction and the release of pigment from the cytoplasm [35]. Color change is associated with water loss, breakdown of cell structure, and degradation of nutrients [36]. Color saturation (C*) and hue angle (h°) values were used to monitor the changes in chlorophyll pigments during the processing of Tartary buckwheat sprouts. Compared with the CK group, h° increased significantly in the CP, OM, β-CD, and US groups, indicating a stronger green hue [20]

The rehydration rate of different pretreatments increased with the increase in drying temperature. As the hot air drying temperature increased, the rehydration characteristics of the control group and the citric acid group gradually decreased. However, the other groups showed a trend of first increasing and then decreasing, with the best rehydration at 60 °C. At 60 °C, the rehydration characteristics of buds in the CP, OM, BC, β-CD, and US groups were increased by 32.5%, 40.3%, 12.9%, 70.9%, and 56.7%, respectively, compared to the CK group. It can be concluded that different treatment groups can improve the rehydration characteristics of Tartary buckwheat sprouts, and β-cyclodextrin has the best effect. This may be because of the inclusion structure of beta-cyclodextrin itself, which causes water to be absorbed more easily by inclusion. Ultrasound can cause the formation of micropores in the external tissues of Tartary buckwheat sprouts, which improves the water absorption capacity during the rehydration process [9]. Additionally, during the osmotic process, the sample undergoes osmotic pressure, changing the microchannels of cell tissues and facilitating water flow, thus improving rehydration [37].

### 3.3. Effects of Hot Air Drying on Physical Characteristics of Tartary Buckwheat Sprouts under Different Pretreatment Conditions

**Figure 2:**
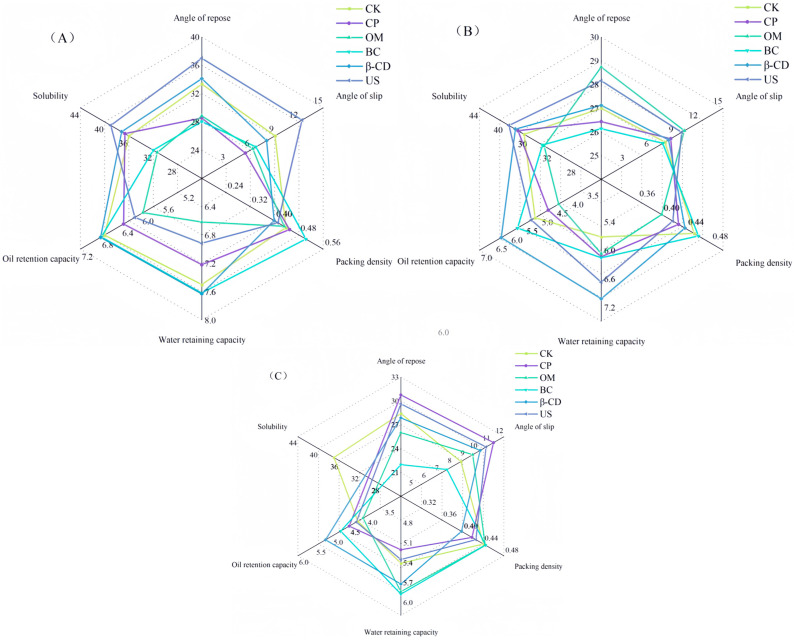
Effects of hot air drying on physical characteristics of Tartary buckwheat sprouts under different pretreatment conditions. Note: (**A**) Hot air drying temperature: 50 °C; (**B**) Hot air drying temperature:60 °C; (**C**) Hot air drying temperature: 70 °C.

The physical characteristics of Tartary buckwheat sprout powder under different hot air drying treatments are shown in Figure 2. The angle of rest and the angle of slip can reflect the fluidity of the powder. Generally speaking, the smaller the angle of rest and the angle of slip, the better the fluidity of the powder [38]. The resting angle of Tartary buckwheat sprout powder in the CK, US, β-CD, and CP groups decreased first and then with the increase in hot air drying temperature, indicating better fluidity at 60 °C. However, under the same temperature conditions, the resting angle of samples in group BC is the lowest. This may be due to the tissue destruction during the blanching process, leading to significant nutrient loss in the sample and resulting in low viscosity [13]. The higher resting angles of US, β-CD, and OM groups may be attributed to the ease with which the tissue grinding process absorbs moisture, resulting in poor sample fluidity. The water and oil holding capacity of Tartary buckwheat sprout powder decreased gradually with the increase in hot air temperature. It may be that high temperatures damage the cell structure and cause cell damage; thus, the binding force on water molecules and oil is weakened [13]. At the same temperature, the water and oil holding capacity of the β-CD group were the highest at 7.63 and 6.23 g·g^−1^, respectively.

The packing density varied at different hot air drying temperatures across different treatments, potentially influenced by structural changes resulting from varying hot air drying parameters [39]. As the hot air drying temperature increased, the bulk density of group CK initially increased and then decreased. At the same hot air temperature, the packing density of sprout powder in group BC was higher than that in group CK, with the highest packing density of 0.50 × 10^3^ kg/cm^3^ observed at 50 °C. Apart from blanching, the solubility of Tartary buckwheat sprout powder increased gradually with higher hot air temperatures across all treatment methods. The highest solubility of the US group at 70 °C was 40.05%. There was no significant difference between the CP and β-CD groups (*p* > 0.05). Compared with the CK group, the US, CP, and β-CD treatments can improve the solubility of the sample.

### 3.4. Effects of Hot Air Drying on Nutrient Composition of Tartary Buckwheat Sprouts under Different Pretreatment Conditions

As shown in Table 3, the cellulose content of Tartary buckwheat sprouts first decreased and then increased with the increase in hot air temperature. Compared to the CK group, the cellulose content in all treatment groups was significantly higher than that in the control group at the same temperature (*p* < 0.05). At 70 °C, the US, OM, and BC groups showed the highest cellulose content at 34.49%, 32.58%, and 33.99%, respectively, with no significant difference observed among them. Regarding reducing sugar, at a hot air temperature of 50 °C, the β-CD and OM groups exhibited higher levels of reducing sugars at 38.56 mg/g and 39.03 mg/g, respectively, with no significant difference between them. Both were significantly higher than those in the CK group (*p* < 0.05). However, the BC group experienced a significant loss of reducing sugars. These sugars tend to react with α-amino acids of nitrogen compounds, altering the color of Tartary buckwheat sprouts and resulting in reduced sugar content [40].

As the hot air drying temperature increased, the soluble protein content in all treatment groups decreased gradually, except in the BC group. At 50 °C, the β-CD group showed the highest soluble protein content at 15.16 mg/g, making a 10.89% increase compared to the CK group, with no significant difference between the CP and OM groups (*p* > 0.05). The BC treatment group experienced the most significant loss of soluble protein, likely due to the high temperature conditions during the blanching process, which led to nutrient loss in the sprouts. In terms of starch content in sprouts, under the drying temperature of 50 °C, the highest content in US group was 45.83 mg/g, 36.2% higher than that in the control group. At the drying temperature of 70 °C, the lowest starch content of group BC was 17.19 mg/g. High temperatures induce the gelatinization of starch in Tartary buckwheat sprouts, thereby emphasizing the significance of drying temperature and duration as critical factors affecting their starch content [41].

Chlorophyll in green plants is highly unstable and prone to decomposition into olive fuscin and phytoseiulus by light, heat, acid, alkali and oxygen [42]. Therefore, it is observed that under the condition of 70 °C, the total chlorophyll content of Tartary buckwheat sprouts is the lowest. At 50 °C, the highest content of the chlorophyllin β-CD group was 3.67 mg/g, which was 37.4% higher than that in control group. The highest content of chlorophyll b in CP group was 1.28 mg/g. The total chlorophyll content of CP and β-CD groups was the highest, and there was no significant difference between them (*p* < 0.05), and it decreased gradually with increasing temperature.

### 3.5. Effect of Different Pretreatment on Active Ingredients of Tartary Buckwheat Sprouts Dried by Hot Air

**Figure 3:**
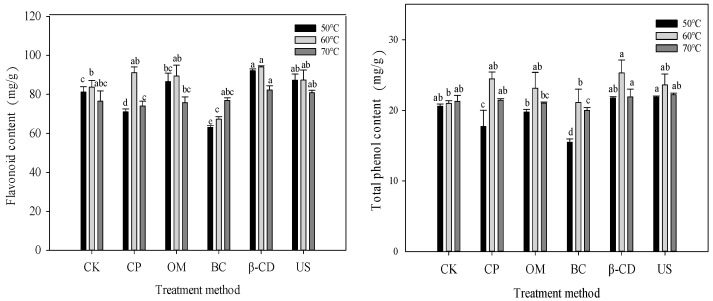
Effect of different pretreatment on active ingredients of Tartary buckwheat sprouts dried by hot air. Note: Different lowercase letters indicate significant statistical differences (*p* < 0.05).

As shown in Figure 3, with the increase in temperature, the contents of total flavonoids and total phenols first increased and then decreased. It was concluded that at 60 °C, the contents of total flavonoids in CP, OM, β-CD and US groups were 91.12 mg/g, 89.44 mg/g, 94.12 mg/g and 87.34 mg/g, respectively, which were increased by 8.76%, 6.76%, 12.34% and 4.25% compared with CK group, respectively. The content of total flavonoids in BC group was the lowest. In contact with water during heat treatment, rutin will be converted into quercetin under the action of enzymes, which will lead to a reduction in flavonoid content. For total phenol content, the highest total phenol content in β-CD group was 25.30 mg/g at 60 °C. At 50 °C, the total phenol content of BC group was at least 15.49 mg/g. According to the test results, citric acid color protection, sodium chloride penetration, β-cyclodextrin treatment and ultrasonic treatment can only reduce the loss of active ingredients in the hot air drying process of Tartary buckwheat sprouts to a certain extent, and β-cyclodextrin treatment has the best effect.

### 3.6. Effect of Hot Air Drying on Antioxidant Capacity of Tartary Buckwheat Sprouts under Different Pretreatment Conditions

**Figure 4:**
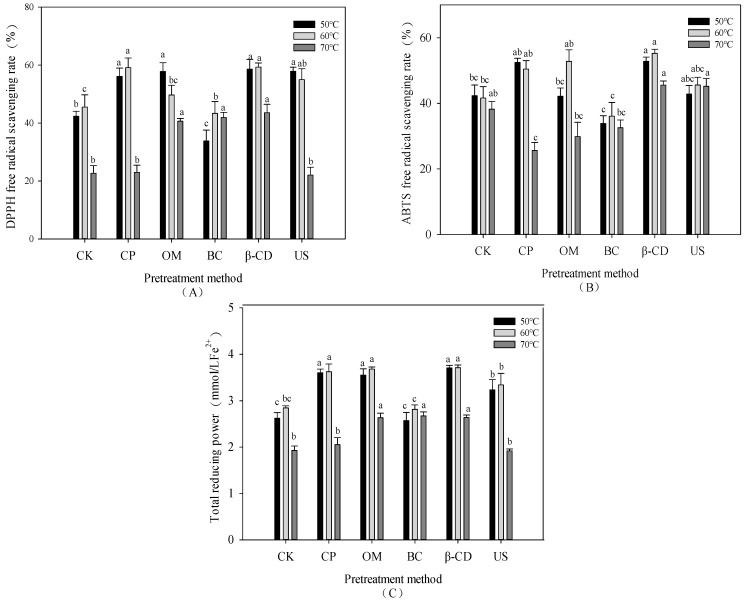
Effect of hot air drying on antioxidant capacity of Tartary buckwheat sprouts under different pretreatment conditions: (**A**) DPPH free radical scavenging rate; (**B**) ABTS free radical scavenging; (**C**) Total reducing power. Note: Different lowercase letters indicate significant statistical differences (*p* < 0.05).

The ABTS free radical scavenging ability of Tartary buckwheat sprouts under different hot air drying methods is shown in Figure 4B. It can be seen that BC treatment reduced the ABTS free radical scavenging ability of Tartary buckwheat sprouts. With the increase in hot air drying temperature, the ABTS free radical scavenging ability of CK group decreased gradually, but the difference was not significant (*p* > 0.05). Under hot air temperatures of 50 °C and 60 °C, CP, OM, β-CD and US groups were higher than the CK group. Under the condition of hot air drying at 60 °C, the highest ABTS free radical scavenging rate of β-CD group was 55.23%. Phenols are the most important phytochemical contributions to the antioxidant capacity of fruits and vegetables [43]. Combined with Table 3, it can be seen that there is a high correlation between the contents of total flavonoids, total phenols and antioxidants. On the whole, the Tartary buckwheat sprouts under the condition of hot air drying at 60 °C have strong antioxidant capacity. At 50 °C and 60 °C, the DPPH free radical scavenging ability of CP, OM, β-CD and US groups was significantly increased compared with the CK group (*p* < 0.05). The effect of each pretreatment on the hot air drying process of Tartary buckwheat sprouts is different, citric acid can improve the antioxidant effect, and β-cyclodextrin has the antioxidant effect. Both penetration and ultrasound reduce the non-enzymatic browning process of the sprouts during drying and reduce the drying time by improving the microscopic channels of the sample tissue [9]. At 60 °C, the highest DPPH free radical clearance rate was 59.32% in β-CD group, which was not significantly different from that in the 50 °C group. At 70 °C, the antioxidant capacity of the OM, BC and β-CD groups improved. It can be seen that the effect of different treatment groups under different hot air drying temperatures, and the hot air drying temperature has a greater influence on the active components of Tartary buckwheat sprouts.

As hot air temperatures increased, the total reducing power of all treatment groups initially increased and then decreased. There was no significant difference in total reducing power between treatment groups at 50 °C and 60 °C (*p* > 0.05). Compared to the CK group, the CP, OM, β-CD, and US groups increased the total reducing power of the sprouts. Under the condition of hot air drying at 60 °C, the β-CD group exhibited the highest total reducing power of 3.71 mmol/L Fe^2+^, which was 1.31 times that of the CK group. There was no significant difference between the CP group and the OM group. In conclusion, high temperature drying can reduce the antioxidant capacity of Tartary buckwheat sprouts, which is highly correlated with the total flavonoids and total phenols of the sprouts.

### 3.7. Clustering Heat Map Analysis

**Figure 5:**
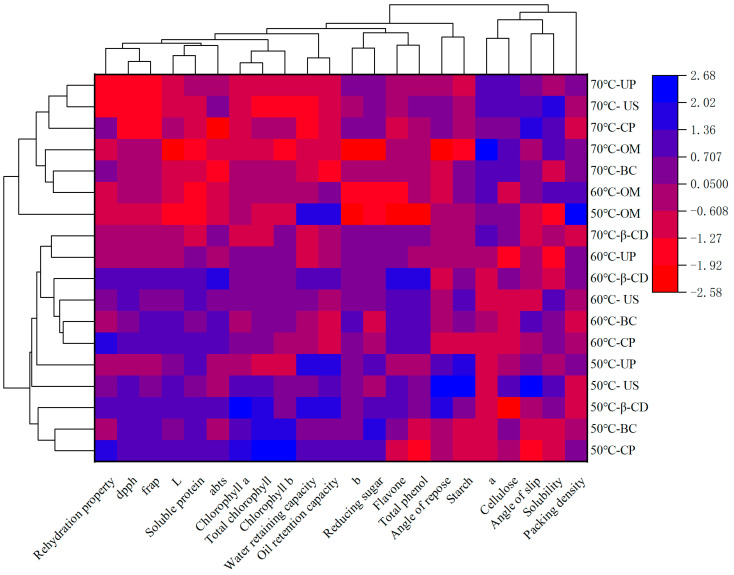
Clustering heat map analysis.

Figure 5 shows the heat map clustering analysis of various data under different hot air drying methods. From the figure, we can see the difference in classification among various hot air drying methods and the correlation between the expression of various indicators. All hot air drying methods can be divided into two categories. The first group includes CP, OM, β-CD, CK, and US under the conditions of hot air drying at 70 °C, as well as OM at 50 °C and 60 °C. The second group was the β-CD group at 70 °C and the pretreatment group at 50 °C and 60 °C. It is evident that the effects at 50 °C and 60 °C are similar. Furthermore, the contents of rehydration, L*, total flavonoids, total phenols, ABTS, DPPH, FRAP, and other antioxidant indexes were expressed at a high level under hot air drying at 60 °C. The content of starch and cellulose was low. At 50 °C, chlorophyll, water holding capacity, oil holding capacity, and other indexes had higher expression. Therefore, under the condition of hot air drying at 60 °C, the active ingredients and antioxidants of Tartary buckwheat sprouts have higher advantages, and the contents of total flavonoids and total phenols in the β-CD group are the highest.

## 4. Conclusions

The study demonstrated that as the drying temperature increased from 50 °C to 70 °C, the drying time of Tartary buckwheat sprouts decreased, and the dehydration rate increased across all treatment groups. Compared to the control group (CK), treatments with OM, US, and BC effectively reduced the drying time of Tartary buckwheat sprouts at each temperature. The BC and US treatments also enhanced the drying rate. Groups treated with CP, β-CD, and US exhibited higher L* values and lower a* values, indicating a more desirable green color. The CP, OM, BC, β-CD, and US treatments were found to enhance the rehydration of the sprouts. The fluidity of Tartary buckwheat sprout powder in the BC group was superior under hot air drying at various temperatures. Additionally, β-CD and US treatments showed improved water and oil retention, enhancing the solubility of the bud powder. The cellulose content of Tartary buckwheat sprouts initially decreased and then increased with rising drying temperatures, while reducing sugar, soluble protein, and soluble sugar exhibited a declining trend. The CP, OM, β-CD, and US treatments notably increased the scavenging power and total reducing power of ABTS and DPPH free radicals. Notably, β-CD was found to have the most significant effects in reducing the loss of active ingredients during the drying process. Overall, the use of various pretreatments along with hot air drying successfully decreased the moisture content in sprouts, minimized the loss of polyphenols, flavonoids, and other active substances, and improved the antioxidant capacity of Tartary buckwheat sprout powder. This approach enables the production of functional Tartary buckwheat sprout powder rich in polyphenols, offering a promising avenue for the value-added processing of vegetables like Tartary buckwheat sprouts. Hot air drying is a commonly used method, but it has the problems of long drying time and high drying temperature. The study of the effect of new drying technologies, such as infrared drying, radio frequency drying, and rotary flash drying, on the drying quality of sprouts is the next stage of research.

## Figures and Tables

**Table 1:** Hot air drying treatment conditions.

Pretreatment Method	Operation Method	Abbreviation
Control check	Without any treatment.	CK
Color-protecting	Immerse in 0.3% citric acid solution; the ratio of solid to liquid is 1:20. Soak at room temperature for 5 min, then drain and wipe the surface water.	CP
Osmosis	Immerse in a beaker containing 3% NaCl solution for 30 min with a solid-liquid ratio of 1:20. Soak at room temperature for 30 min, then drain and wipe the surface moisture.	OM
Blanching	Immersed in a beaker containing distilled water. The ratio of material to liquid is 1:20. Blanch at 85 °C for 30 s, then drain and quickly cool with cold water; wipe the surface moisture.	BC
β-cyclodextrin	Immerse in 1.5% β-cyclodextrin solution with a ratio of material to liquid of 1:20, soak at room temperature for 30 min, then drain and wipe the surface moisture.	β-CD
Ultrasound	Immerse in a beaker filled with distilled water, the ratio of material to liquid is 1:20, and treat at 150 W, 40 Hz, at room temperature for 30 min, then drain and wipe the surface water.	US

**Table 2:** Effects of pretreatment on color and rehydration of Tartary buckwheat sprouts after hot air drying.

Drying Temperature (°C)	Method	L*	a*	b*	C*	h°	Rehydration Property
50	CK	64.67 ± 0.77 ^ab^	5.73 ± 0.60 ^b^	17.97 ± 0.26 ^b^	18.87 ± 0.42 ^a^	72.37 ± 1.52 ^c^	4.89 ± 0.31 ^bc^
	CP	66.05 ± 0.55 ^a^	4.44 ± 0.60 ^c^	18.71 ± 0.84 ^a^	19.24 ± 0.77 ^a^	76.65 ± 2.03 ^ab^	6.36 ± 0.59 ^a^
	OM	63.64 ± 0.61 ^c^	4.51 ± 0.44 ^c^	17.77 ± 0.97 ^bc^	18.34 ± 0.83 ^b^	75.73 ± 2.03 ^b^	5.01 ± 0.57 ^bc^
	BC	58.03 ± 0.70 ^de^	9.35 ± 0.64 ^a^	11.23 ± 0.30 ^d^	14.62 ± 0.53 ^d^	50.26 ± 1.83 ^d^	4.33 ± 0.68 ^c^
	β-CD	66.00 ± 0.88 ^a^	3.92 ± 0.36 ^d^	17.48 ± 0.03 ^c^	17.92 ± 0.10 ^c^	77.39 ± 1.12 ^a^	6.16 ± 0.28 ^a^
	US	65.85 ± 0.70 ^a^	4.54 ± 0.50 ^c^	18.11 ± 0.69 ^a^	18.67 ± 0.62 ^b^	75.93 ± 1.74 ^b^	5.36 ± 0.45 ^ab^
60	CK	62.53 ± 0.14 ^d^	6.43 ± 0.88 ^b^	17.64 ± 0.32 ^c^	18.79 ± 0.16 ^c^	70.00 ± 2.80 ^c^	4.15 ± 0.30 ^d^
	CP	65.48 ± 0.88 ^a^	5.60 ± 0.57 ^b^	17.95 ± 0.38 ^c^	18.81 ± 0.19 ^c^	72.68 ± 2.00 ^ab^	5.19 ± 0.37 ^bc^
	OM	65.39 ± 0.81 ^a^	5.83 ± 0.47 ^b^	19.06 ± 0.08 ^a^	19.94 ± 0.09 ^a^	73.04 ± 1.33 ^a^	5.65 ± 0.59 ^b^
	BC	60.02 ± 2.31 ^de^	10.18 ± 1.33 ^a^	12.21 ± 0.85 ^d^	15.95 ± 0.23 ^d^	50.26 ± 5.59 ^d^	4.68 ± 0.21 ^cd^
	β-CD	65.78 ± 1.09 ^a^	5.63 ± 0.62 ^b^	18.30 ± 0.38 ^b^	19.15 ± 0.41 ^b^	72.95 ± 1.80 ^ab^	6.42 ± 0.43 ^a^
	US	64.04 ± 0.55 ^ab^	5.75 ± 0.60 ^b^	18.48 ± 0.31 ^b^	19.36 ± 0.26 ^b^	72.74 ± 1.85 ^ab^	6.50 ± 0.13 ^a^
70	CK	59.61 ± 1.25 ^d^	10.44 ± 0.79 ^c^	17.06 ± 0.17 ^ab^	20.01 ± 0.36 ^a^	58.58 ± 2.06 ^b^	3.64 ± 0.44 ^c^
	CP	62.23 ± 1.01 ^c^	8.09 ± 0.56 ^d^	17.81 ± 0.33 ^a^	19.56 ± 0.46 ^b^	65.61 ± 1.35 ^a^	5.42 ± 0.53 ^a^
	OM	59.78 ± 0.62 ^d^	11.69 ± 0.51 ^b^	16.65 ± 0.61 ^c^	20.35 ± 0.24 ^a^	54.94 ± 2.15 ^c^	5.14 ± 0.44 ^ab^
	BC	56.41 ± 1.11 ^f^	14.61 ± 0.61 ^a^	10.62 ± 0.75 ^e^	18.08 ± 0.26 ^c^	36.02 ± 2.95 ^d^	4.33 ± 0.37 ^bc^
	β-CD	61.52 ± 0.82 ^cd^	9.97 ± 0.84 ^c^	17.28 ± 0.49 ^a^	19.97 ± 0.32 ^ab^	60.04 ± 2.62 ^b^	5.07 ± 0.39 ^ab^
	US	59.77 ± 0.79 ^d^	11.42 ± 0.54 ^b^	15.62 ± 0.68 ^d^	19.36 ± 0.60 ^b^	53.83 ± 1.84 ^c^	3.71 ± 0.30 ^c^

Note: Different lowercase letters indicate significant statistical difference between different groups (in the same column) (*p* < 0.05).

**Table 3:** Effects of hot air drying on nutrient composition of Tartary buckwheat sprouts under different pretreatment conditions.

Drying Temperature (°C)	Method	Soluble Protein (mg/g)	Reducing Sugar (mg/g)	Starch (mg/g)	Cellulose (mg/g)	Chlorophyll a (mg/g)	Chlorophyll b (mg/g)	Total Chlorophyll (mg/g)
50	CK	13.68 ± 0.47 ^b^	37.04 ± 1.02 ^b^	33.65 ± 1.02 ^b^	24.72 ± 1.69 ^d^	0.53 ± 0.05 ^c^	0.10 ± 0.02 ^c^	0.63 ± 0.04 ^c^
	CP	14.75 ± 0.85 ^a^	37.42 ± 2.40 ^ab^	21.63 ± 0.92 ^d^	24.68 ± 1.65 ^d^	0.72 ± 0.01 ^a^	0.26 ± 0.01 ^a^	0.98 ± 0.06 ^a^
	OM	14.32 ± 0.47 ^a^	39.03 ± 1.06 ^a^	22.10 ± 0.40 ^d^	30.67 ± 1.03 ^ab^	0.66 ± 0.02 ^b^	0.23 ± 0.03 ^a^	0.89 ± 0.01 ^ab^
	BC	5.57 ± 0.47 ^c^	29.73 ± 2.21 ^c^	27.35 ± 2.94 ^c^	28.93 ± 0.84 ^c^	0.53 ± 0.02 ^c^	0.10 ± 0.01 ^c^	0.63 ± 0.03 ^c^
	β-CD	15.16 ± 0.42 ^a^	38.56 ± 1.96 ^a^	30.73 ± 2.45 ^c^	15.73 ± 2.57 ^e^	0.73 ± 0.03 ^a^	0.16 ± 0.04 ^ab^	0.90 ± 0.08 ^ab^
	US	12.87 ± 0.76 ^b^	34.52 ± 0.58 ^b^	45.83 ± 1.93 ^a^	32.63 ± 1.27 ^a^	0.64 ± 0.02 ^b^	0.18 ± 0.04 ^ab^	0.82 ± 0.02 ^b^
60	CK	11.67 ± 0.45 ^c^	32.51 ± 2.36 ^b^	26.88 ± 3.50 ^c^	17.98 ± 0.84 ^d^	0.58 ± 0.08 ^a^	0.18 ± 0.02 ^a^	0.76 ± 0.04 ^a^
	CP	13.48 ± 11.94 ^b^	33.56 ± 0.59 ^b^	20.81 ± 1.06 ^d^	23.03 ± 1.68 ^bc^	0.58 ± 0.05 ^a^	0.15 ± 0.06 ^ab^	0.73 ± 0.02 ^a^
	OM	11.95 ± 0.68 ^c^	31.83 ± 2.58 ^b^	31.54 ± 1.45 ^b^	19.94 ± 0.49 ^cd^	0.56 ± 0.04 ^a^	0.17 ± 0.02 ^a^	0.73 ± 0.04 ^a^
	BC	5.20 ± 0.52 ^d^	29.26 ± 1.25 ^c^	31.90 ± 1.80 ^b^	21.68 ± 2.65 ^bc^	0.52 ± 0.04 ^a^	0.14 ± 0.01 ^b^	0.67 ± 0.03 ^c^
	β-CD	14.34 ± 0.50 ^a^	36.09 ± 1.48 ^a^	34.93 ± 1.40 ^b^	27.25 ± 0.84 ^a^	0.58 ± 0.03 ^a^	0.18 ± 0.02 ^a^	0.76 ± 0.23 ^a^
	US	13.91 ± 0.13 ^b^	35.64 ± 0.95 ^a^	39.37 ± 2.53 ^a^	23.31 ± 1.75 ^b^	0.57 ± 0.04 ^a^	0.16 ± 0.02 ^a^	0.74 ± 0.05 ^a^
70	CK	8.76 ± 0.65 ^a^	35.97 ± 1.23 ^a^	18.71 ± 4.85 ^c^	31.74 ± 1.29 ^ab^	0.48 ± 0.01 ^b^	0.11 ± 0.01 ^a^	0.60 ± 0.03 ^c^
	CP	7.09 ± 0.30 ^bc^	37.29 ± 1.10 ^a^	27.11 ± 0.53 ^ab^	30.34 ± 2.57 ^bc^	0.51 ± 0.04 ^a^	0.14 ± 0.04 ^a^	0.66 ± 0.03 ^a^
	OM	7.45 ± 0.37 ^b^	32.51 ± 0.89 ^a^	31.43 ± 1.05 ^a^	32.58 ± 2.57 ^ab^	0.53 ± 0.01 ^a^	0.13 ± 0.01 ^a^	0.66 ± 0.01 ^ab^
	BC	6.15 ± 0.68 ^c^	27.12 ± 2.54 ^c^	17.19 ± 0.53 ^c^	33.99 ± 0.84 ^a^	0.50 ± 0.02 ^a^	0.08 ± 0.01 ^a^	0.59 ± 0.01 ^c^
	β-CD	8.00 ± 0.67 ^a^	34.58 ± 1.52 ^b^	24.78 ± 0.92 ^b^	28.09 ± 1.69 ^c^	0.47 ± 0.02 ^b^	0.18 ± 0.02 ^a^	0.65 ± 0.01 ^ab^
	US	8.07 ± 0.74 ^a^	35.43 ± 2.44 ^a^	23.96 ± 2.63 ^b^	34.49 ± 0.89 ^a^	0.49 ± 0.01 ^b^	0.08 ± 0.01 ^a^	0.57 ± 0.02 ^d^

Note: Different lowercase letters indicate significant statistical difference between different groups (in the same column) (*p* < 0.05).

## Data Availability

The original contributions presented in the study are included in the article, further inquiries can be directed to the corresponding authors.
